# P-809. Evaluating the Usage, Safety, and Accuracy of Vancomycin Dosing Practices at VA Eastern Colorado Health Care System (VA ECHCS)

**DOI:** 10.1093/ofid/ofae631.1001

**Published:** 2025-01-29

**Authors:** Caitlin Hart, James Christoffersen, Catherine Scott

**Affiliations:** VA Eastern Colorado Health Care System, Evergreen, Colorado; VA Eastern Colorado Health Care System, Evergreen, Colorado; VA Eastern Colorado Health Care System, Evergreen, Colorado

## Abstract

**Background:**

Vancomycin is a glycopeptide antibiotic used as a mainstay of therapy for gram positive bacterial infections, including MRSA (methicillin-resistant *staphylococcus aureus*). Vancomycin requires pharmacokinetic calculations to determine dosing and close therapeutic drug monitoring to achieve goal trough between 15-20 mg/L (AUC/MIC goal between 400-600 mg ■h/L) and prevent nephrotoxicity. The purpose of this study is to assess our current vancomycin usage patterns, including empiric dosing accuracy & safety outcomes to help inform a decision whether a change to our current practice is indicated.

**Methods:**

This retrospective, observational cohort study evaluated 117 vancomycin trough levels from 63 patients who received at least two doses of vancomycin between August 1, 2022 and July 31, 2023. Patients were excluded if they received hemodialysis or were dosed by random levels in the setting of severe renal dysfunction. Collected demographics included age, sex, weight, height, BMI, indication for vancomycin, dosing, trough levels, source of infection, MRSA nares, concomitant nephrotoxic medications, spinal cord injury diagnosis, serum creatinine, and cystatin C. Estimated AUC was calculated using VancoPK.com based on the reported trough using patient specific pharmacokinetic parameters. This study is exempt from IRB approval.

**Results:**

A total of 58 trough levels from 49 patients were included. The majority of patients were male (87.7%) with an average age 61±13 years old. Out of the 58 trough levels evaluated, 20 (41%) were at goal. Of the levels not a goal 27 (71%) were subtherapeutic, and 11 (29%) were supratherapeutic. The estimated AUC was at goal for 35 (60%) of the 58 total troughs. Of the 27 patients who had a subtherapeutic trough 21 (77%) had an AUC at goal, and 14 of them increased the dose despite having a therapeutic AUC. Only 1 patient (2%) developed possible vancomycin associated AKI (acute kidney injury).

**Conclusion:**

A higher number of patients were within therapeutic range when evaluating AUC compared to trough. The low incidence of vancomycin associated AKI was likely due to low frequency of supratherapeutic troughs in this study. Further analysis of these results will be used to identify opportunities for education and improvement of our current vancomycin dosing process.

**Disclosures:**

**All Authors**: No reported disclosures

